# Are children and adolescents living with HIV in Europe and South Africa at higher risk of SARS-CoV-2 and poor COVID-19 outcomes?

**DOI:** 10.1017/S0950268825000135

**Published:** 2025-02-21

**Authors:** The European Pregnancy and Paediatric Infections Cohort Collaboration (EPPICC) SARS-CoV-2 Antibody Study Group

**Keywords:** COVID-19, HIV/AIDS, serology

## Abstract

Children, adolescents, and young people living with HIV (CALWHIV), including those in resource-limited settings, may be at increased risk of severe acute respiratory syndrome coronavirus 2 (SARS-CoV-2) infection, poorer coronavirus disease 2019 (COVID-19) outcomes, and multisystem inflammatory syndrome (MIS). We conducted a repeat SARS-CoV-2 seroprevalence survey among CALWHIV in Europe (*n* = 493) and South Africa (SA, *n* = 307), and HIV-negative adolescents in SA (*n* = 100), in 2020–2022. Blood samples were tested for SARS-CoV-2 antibody, questionnaires collected data on SARS-CoV-2 risk factors and vaccination status, and clinical data were extracted from health records. SARS-CoV-2 seroprevalence (95% CI) was 55% (50%–59%) in CALWHIV in Europe, 67% (61%–72%) in CALWHIV in SA, and 85% (77%–92%) among HIV-negative participants in SA. Among those unvaccinated at time of sampling (*n* = 769, 85%), seroprevalence was 40% (35%–45%), 64% (58%–70%), and 81% (71%–89%), respectively. Few participants (11% overall) had a known history of SARS-CoV-2-positive PCR or self-reported COVID-19. Three CALWHIV were hospitalized, two with COVID-19 (nonsevere disease) and one young adult with MIS. Although SARS-CoV-2 seroprevalence was high across all settings, even in unvaccinated participants, it was broadly comparable to general population estimates, and most infections were mild/asymptomatic. Results support policy decisions excluding CALWHIV without severe immunosuppression from high-risk groups for COVID-19.

## Introduction

People living with HIV (PLWHIV) may be at a greater risk of severe outcomes following coronavirus disease 2019 (COVID-19, caused by severe acute respiratory syndrome coronavirus 2 (SARS-CoV-2)) than people who are HIV-negative, although the extent of and reasons for any causal relationship remain unclear [1, 2]. The impact of factors such as HIV viral load (VL), CD4 count, antiretroviral therapy (ART), and comorbidities is incompletely understood [1, 2]. These factors may vary between settings, for example, depending on country income status, meaning that collection of comparable data from multiple countries is essential.

Data on severity of SARS-CoV-2 infection in children, adolescents, and young people living with HIV (CALWHIV) are limited, although two European studies reported only mild disease in this group [3, 4], consistent with data on HIV-negative children [5]. A comparison between CALWHIV and HIV-negative children in South Africa (SA) found no association between HIV and SARS-CoV-2 mortality, although precision was limited by the reassuringly small number of deaths [6].

While polymerase chain reaction (PCR) tests detect current SARS-CoV-2 infection, serological tests detect antibodies which indicate either previous infection or vaccination. Antibodies against the SARS-CoV-2 nucleoprotein (anti-N antibodies) are an index of natural infection, whereas those against the spike protein (anti-S antibodies) indicate previous infection or vaccination with currently available vaccines. Conversely, absence of antibodies indicates that either an individual has never been infected with or vaccinated against SARS-CoV-2, or that any antibody response to infection or vaccination was below the assay limit of detection or has waned over time. SARS-CoV-2 serological tests have high sensitivity and specificity, although sensitivity varies between tests and wanes over time since infection [7].

Seroprevalence data can be compared to clinical data to infer the severity of SARS-CoV-2 infection. Although the extent of protection conferred by infection with previous variants is unclear, these data can inform vaccine policy, for example, whether CALWHIV should be prioritized for vaccination or messaging to promote vaccine uptake. SARS-CoV-2 seroprevalence data in CALWHIV are limited. A study in Mozambique reported SARS-CoV-2 seroprevalence as 36% among 90 unvaccinated adolescents living with HIV compared to 49% among 450 HIV-negative participants in November 2022 [8], with evidence that seroprevalence was lower among adolescents living with HIV on unadjusted, but not adjusted, analysis.

In this study, we estimate the prevalence of SARS-CoV-2 antibody in CALWHIV in Europe and SA, and a comparison group of HIV-negative adolescents and young people in SA, and describe how this changed over time, overall and by age group and region.

## Methods

We carried out a repeat SARS-CoV-2 seroprevalence study among CALWHIV enrolled through established cohorts within the European Pregnancy and Paediatric Infections Cohort Collaboration (EPPICC) and the Cape Town Adolescent Antiretroviral Cohort (CTAAC). EPPICC is a network of cohorts of CALWHIV in routine pediatric HIV care in Europe and Thailand [9]; a subset of six cohorts in five countries participated in this study (Belgium, Greece, two cohorts in Spain, Ukraine, and the UK). Some cohorts include follow-up data after transfer to adult HIV care. CTAAC is a longitudinal cohort study of adolescents living with HIV established on ART and a comparison group from the same communities of age-matched HIV-negative adolescents in Cape Town, SA [10].

### Participants and study procedures

Participants aged <25 years and in follow-up in these cohorts were invited to take part in this study. CALWHIV were eligible if diagnosed with HIV aged <18 years. At the start of the study (October 2020), participation in a SARS-CoV-2 vaccine trial or receipt of a vaccine at baseline were exclusion criteria; the latter criterion was removed in May 2021 as approved vaccines became increasingly available.

Venous blood samples and participant data were collected during routine clinic or study visits at two time points ~6 months apart (allowable range 3–13 months). Participants (or parents/guardians for children) completed questionnaires including information on COVID-19 (physician-diagnosed with or without a positive test, or self-reported) at each visit. Additional details on SARS-CoV-2 vaccination status were added from March 2021.

In some clinics, previous test results from routine SARS-CoV-2 antibody screening or stored samples from routine visits (after 1 March 2020 and ≥4 months before enrollment) were used as the baseline sample. Questionnaires were completed referring to the time of sample collection. All samples were tested for SARS-CoV-2 antibodies using locally available serological assays. Results for anti-S IgG were preferred as these antibodies have the longest half-life [11, 12]; other results (e.g. for IgM and/or anti-N) were also accepted. Results were reported as positive, negative, or indeterminate as per manufacturers’ instructions.

At each visit, data were extracted from clinic records, including demographics, HIV (clinical, laboratory, and ART data; CD4 counts and HIV VLs measured up to 6 months before to one month after the sample date), comorbidities at the time of the test, dates of COVID-19 diagnoses (SARS-CoV-2-positive PCR and/or hospitalization with symptoms consistent with COVID-19 according to WHO definition (Supporting Information)) and multisystem inflammatory syndrome in children (MIS-C, based on the WHO definition [13]), and dates and details of SARS-CoV-2 vaccination. For participants with documented COVID-19 or MIS-C diagnosis, clinicians were asked to provide further details using case report forms based on WHO/ISARIC forms, including reporting of severity as defined by WHO at the time [14] (Supporting Information), although this was not possible in SA. The MIS-C case definition applies to 0–19 year-olds; we did not systematically collect data on post-SARS-CoV-2 inflammatory syndromes in older participants.

Information on the dominant variant in each setting for each calendar quarter was based on publicly available data from the Global Initiative on Sharing All Influenza Data (GISAID) [15]; a variant was considered dominant from the first month in which it accounted for ≥50% of reports.

### Statistical analysis

The original target sample size was 1150 (650 in Europe, 500 in SA). Assuming a seroprevalence of 10% [16], this would produce a 95% confidence interval of ±2% in Europe and ±3% in SA. As reduced clinic visits during the pandemic affected enrollment, we revised the target sample size to 950 in November 2021, with minimal impact on precision.

Participant characteristics at baseline were summarized as frequency/percentage and median [interquartile range]. As our primary outcome, we summarized the percentage of participants with at least one SARS-CoV-2 antibody positive blood sample, overall and by sex, age group (<15 vs. ≥15 years), presence of any comorbidity and geographical setting (Europe and SA). Within Europe we further stratified as UK, Ukraine, and ‘rest of Europe’ (Belgium, Greece, and Spain) based on sample size. In SA, we stratified by HIV status. Vaccine coverage is presented as the percentage of samples each quarter that were from participants who had received at least one vaccine dose, among those with known vaccination status.

We also present the percentage of tests that were positive, with exact 95% CIs, by calendar quarter in Europe (overall and for UK, Ukraine, and rest of Europe) and SA (by HIV status), where the denominator was ≥10.

To assess seroprevalence due to infection (rather than vaccination), we carried out two separate analyses restricted to (1) samples from participants reported as unvaccinated at the time of blood sampling and (2) tests for N-antibodies.

To assess antibody status among vaccinated participants, we estimated the percentage with a positive S-antibody result on their first test after vaccination. Finally, among all participants who were seropositive on their first test, we estimated the percentage who reverted to seronegative on their second.

## Results

Between October 2020 and April 2022, 906 participants were enrolled across six countries, providing 1679 serology test results. Eighteen results for 16 participants were indeterminate; three of these participants had no valid results and were excluded, whereas 13 had one remaining valid result. Therefore 1661 tests for 903 participants were included in analyses (for types of tests see Supplementary Table S1). Samples were taken between May 2020 and July 2022 (Supplementary Figure S1). Among 758 participants with two test results, the median time between samples was 192 days [IQR 176–259]. The time between samples was <90 days for eight participants and >395 days for 28; although outside the recommended range, these were retained in analyses. 898/903 (99%) participants completed at least one questionnaire and 657/758 (87%) of those with two tests completed two questionnaires.

### Participant characteristics at baseline

Most participants were enrolled in SA (410/903, 45%), UK (202/903, 22%), or Ukraine (160/903, 18%). 800/903 (89%) were CALWHIV; 103 (11%) were HIV-negative participants in SA (Table 1). Median age at enrollment was 15 years [IQR 12–18] in Europe, 19 [IQR 17–20] among CALWHIV in SA, and 17 [IQR 16–19] among HIV-negative participants in SA. Approximately half of participants were female. Details of comorbidities are given in Supplementary Table S2. Two participants reported enrollment in a vaccine trial during the study (one each in UK and SA).

**Table 1.** Participant characteristics at enrollment to the study

Characteristic		Median [IQR] or *n* (%)		
		Europe (*N* = 493)	SA, HIV+ (*N* = 307)	SA, HIV− (*N* = 103)
Cohort	South Africa	—	307 (100)	103 (100)
	United Kingdom	202 (41)	—	—
	Ukraine	160 (32)	—	—
	Spain	91 (18)	—	—
	Greece	21 (4)	—	—
	Belgium	19 (4)	—	—
Sex, female		263 (53)	161 (52)	52 (50)
Age, years (*n* = 492, 307, 103)		15 [12–18]	19 [17–20]	17 [16–19]
Any comorbidity		53 (11)	6 (2)	1 (1)
Perinatally acquired HIV (*n* = 469, 307)^a^		462 (99)	307 (100)	—
Age at HIV diagnosis, years (*n* = 467, 307)		1.2 [0.3–3.7]	0 [0–0]	—
Initiated ART before enrollment (*n* = 455, 307)		451 (99)	307 (100)	—
Age at ART start, years (*n* = 453, 307)		2.1 [0.4–5.9]	4.0 [1.9–7.0]	—
CD4 count, cells/μL (*n* = 424, 296)^b^		739 [553–960]	533 [374–690]	—
CD4 count <350 cells/μL (*n* = 424, 296)^b^		22 (5)	66 (22)	—
Undetectable viral load^c^ (*n* = 465, 297)		396 (85)	184 (62)	—
Undetectable viral load^d^ (*n* = 465, 297)		441 (95)	226 (76)	—

a Reported as vertical acquisition or with unknown mode of acquisition diagnosed before age 10 years.

b 88% of baseline CD4 measurements were taken on the day of the baseline serology test.

c <50 copies/mL or below lower limit of detection. 94% of baseline VL measurements were taken on the day of the baseline serology test.

d <1000 copies/mL or below lower limit of detection.

Almost all participating CALWHIV had initiated ART before enrollment. In Europe, 5% of CALWHIV had a CD4 count <350 cells/μL versus 22% in SA (2% and 9% were severely immunocompromised with CD4 <200 cells/μL). 85% of CALWHIV in Europe and 62% in SA were virologically suppressed <50 copies/mL (with 95% and 76% suppressed <1000 copies/mL).

### COVID-19 disease and vaccination at baseline and follow-up

At the time of enrollment, among those with data, 15 participants (all were CALWHIV in Europe) had a previous SARS-CoV-2-positive PCR in their clinic records (3% of CALWHIV in Europe, 2% of participants overall) (Table 2). By the end of follow-up, 5% (44/866) of all participants had a positive PCR recorded. This percentage was highest among CALWHIV in Europe (Table 2). By the end of follow-up, a further 55 (all in Europe) had self-reported COVID-19 but without a recorded positive PCR in their clinic records.

**Table 2.** SARS-CoV-2/COVID-19 infection and vaccination status at baseline and follow-up

			*n*/*N* (%)						
		Baseline				Follow-up			
		Europe	SA, HIV+	SA, HIV−	Total	Europe	SA, HIV+	SA, HIV−	Total
SARS-CoV–2 PCR+		15/482 (3)	0/281 (0)	0/97 (0)	15/860 (2)	40/485 (8)	2/282 (1)	2/99 (2)	44/866 (5)
Self-reported COVID–19, no PCR+^a^						55/440 (13)	0/290 (0)	0/99 (0)	55/829 (7)
Ever received SARS-CoV–2 vaccine		47/463 (10)	0/263 (0)	0/90 (0)	47/816 (6)	146/483 (30)	34/287 (12)	22/100 (22)	202/870 (23)
Any of the above						199/475 (42)	35/280 (13)	24/97 (25)	258/534 (48)

a Self-reported COVID-19 prior to test date not collected at baseline.

At enrollment, 47/463 (10%) participants in Europe with known SARS-CoV-2 vaccine status had been vaccinated, and none in SA. This increased during follow-up, to 202/870 (23%) of all participants (Table 2). Of those vaccinated, 73/202 (36%) had received one dose (including 13 single-dose schedule Janssen vaccines), 114/202 had two doses (56%) and 15/202 (7%) >2 doses. Manufacturer was reported for 259 doses, with the most common being Pfizer/BioNTech (193/259, 75%). Vaccine coverage (≥1 dose) varied by setting and increased over time: among participants providing samples in the final quarter of the study, vaccine coverage reached 50% (95% CI 35%–65%) in Europe, 33% (95% CI 20%–48%) in CALWHIV in SA, and 30% (95% CI 17%–47%) in HIV-negative participants (Supplementary Figure S1). Within Europe, vaccine coverage was markedly lower in Ukraine (6/160, 4%) than in the UK (86/196, 44%) and the remaining European cohorts (54/127, 43%).

In total, among those with data, 199/475 (42%) CALWHIV in Europe, 35/280 (13%) CALWHIV in SA, and 24/97 (25%) HIV-negative participants in SA had any of the following by the end of follow-up: a SARS-CoV-2-positive PCR, self-reported COVID-19, or vaccination (Table 2).

Two participants in Europe were hospitalized with COVID-19 (both PCR-positive), neither of whom was classified as having severe disease. No cases of MIS-C were reported although one 22-year-old participant living with HIV in Europe was diagnosed with Multisystem Inflammatory Syndrome in Adults (MIS-A). They were hospitalized and subsequently discharged. One participant died. This participant was a CALWHIV enrolled in SA, and their death was not related to either COVID-19 or HIV.

### SARS-CoV-2 antibody status

**Figure 1.**
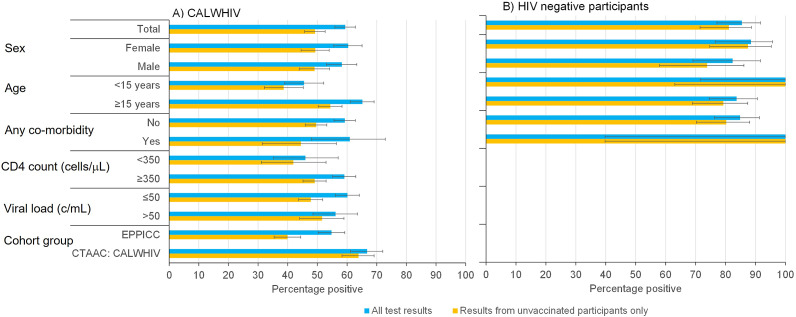
Percentage of (A) CALWHIV- and (B) HIV-negative participants with at least one positive serology test result, overall and by key characteristics. Results are shown based on all tests (blue bars) and on tests from samples taken from unvaccinated participants (yellow bars). Error bars show exact 95% confidence intervals.

In analyses including all participants, irrespective of vaccination status, 55% (95% CI 50-59%) of CALWHIV in Europe, 67% (95% CI 61%–72%) of CALWHIV in SA and 85% (95% CI 77%–92%) of HIV-negative participants were SARS-CoV-2 seropositive on at least one sample (Figure 1, blue bars). When the analysis was restricted to samples taken from unvaccinated participants (*n* = 769), these figures were 40% (95% CI 35%–45%), 64% (95% CI 58%–70%), and 81% (95% CI 71%–89%), respectively (Figure 1, yellow bars).

### Trends in seroprevalence over time and by participant characteristics

**Figure 2.**
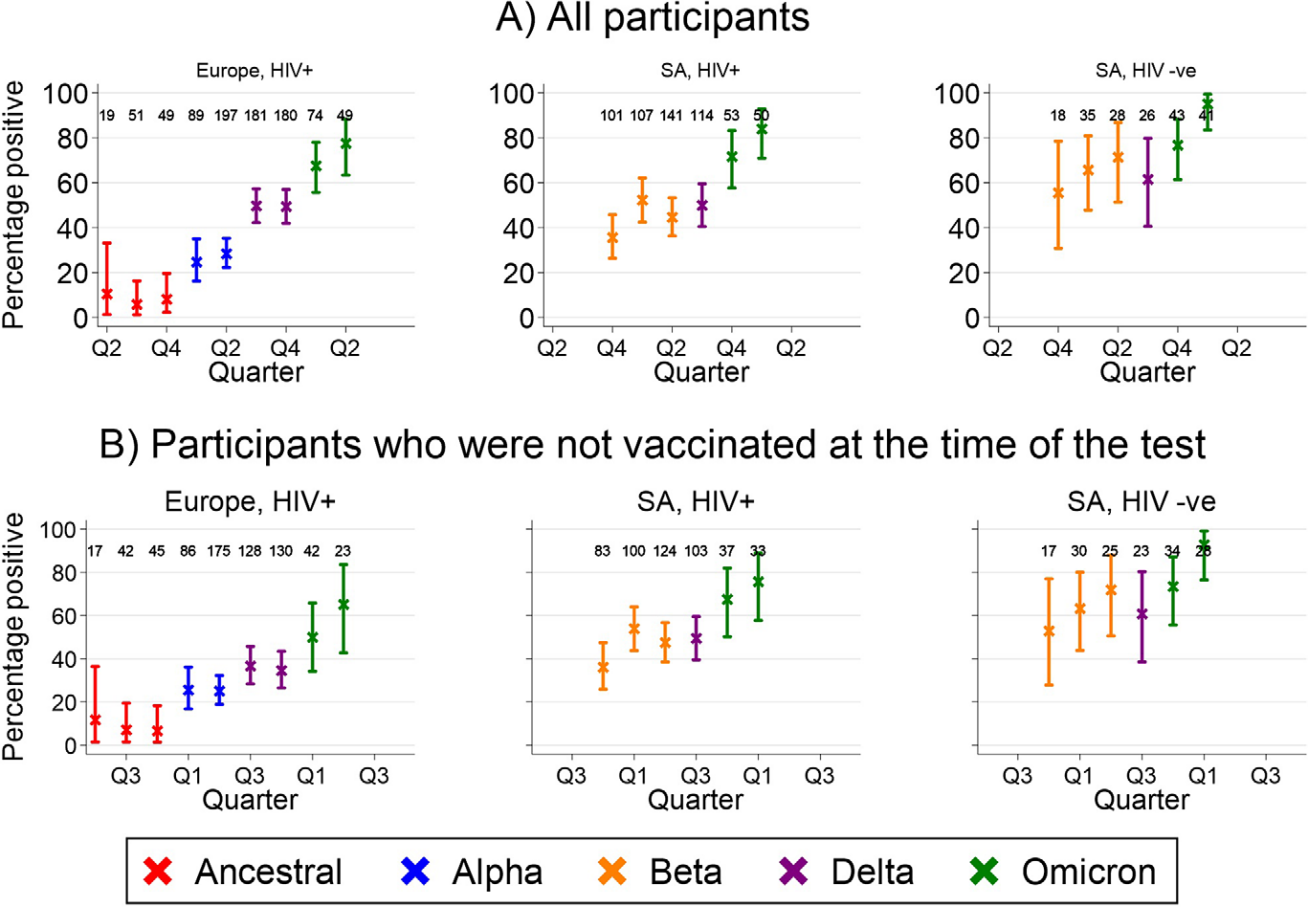
Percentage of serology tests that were positive by cohort group and calendar quarter, overall (top) and among participants who were unvaccinated at the time of the test (bottom). Numbers show the denominator for each estimate.

The percentage of tests that were positive increased over time in all groups (Figure 2). Overall seroprevalence reached 78% (95% CI 63%–88%), 84% (95% CI 71%–93%), and 95% (95% CI 83%–99%) in CALWHIV in Europe, CALWHIV in SA, and HIV-negative participants in SA, respectively. The corresponding figures among unvaccinated participants were 65% (95% CI 43%–84%), 76% (95% CI 58%–89%), and 93% (95% CI 76%–99%). In Europe, seroprevalence in Ukraine varied little over time compared to other countries (Supplementary Figure S3). In all settings, seroprevalence increased following the emergence of new SARS-CoV-2 variants of concern, particularly Omicron.

In CALWHIV, seroprevalence was higher in those aged ≥15 years (65%, 95% CI 61%–69%) than in younger participants (45%, 95% CI 39%–52%) while in HIV-negative participants, seroprevalence was higher in <15 year-olds (100% versus 84% in ≥15 year-olds), although with wide CIs. Among CALWHIV, seroprevalence was higher among participants with baseline CD4 counts ≥350 cells/μL (59% (95% CI 55%–63%)) versus <350 cells/μL (46% (95% CI 35%–57%)), while it did not appear to differ by baseline virological status. Similar patterns were observed when analysis was restricted to samples taken from unvaccinated participants (Figure 1, yellow bars).

### Antibody status among vaccine recipients, seroreversion, and N-antibody results

Among participants known to be vaccinated before blood sampling, 108/119 (91%) were seropositive for S-antibodies on the first test result following vaccination (median 105 days [IQR 35–179] after vaccination). Details of the 11 seronegative participants are shown in Supplementary Table S3.

Among 283 participants (both vaccinated and unvaccinated) who were seropositive on their first test and had two tests, 45 were subsequently seronegative (Supplementary Table S4).

A total of 199 participants had at least one N-antibody result: 160 in Ukraine, 28 in the UK, and 11 in the rest of Europe. Of these, 83/199 (42%) were positive for N-antibodies on ≥1 test, indicating definite infection rather than vaccination.

## Discussion

This is, to our knowledge, the first study to assess SARS-CoV-2 antibody status in a large, geographically diverse sample of CALWHIV. We found a high seroprevalence (78%–84%) of SARS-CoV-2 antibodies by mid-2022 among CALWHIV in Europe and SA, and 65%–76% among participants with no history of SARS-CoV-2 vaccination at the time of the test. Three CALWHIV in Europe were hospitalized: two with COVID-19 (both with nonsevere disease) and one young adult with MIS-A (although we did not systematically collect data on MIS-A). Nonetheless, the lower prevalence of documented or self-reported SARS-CoV-2 infection or COVID-19 disease compared to seroprevalence implies that many infections were asymptomatic or mild, consistent with other analyses of CALWHIV enrolled in EPPICC [3] and elsewhere [4, 8]. The apparent increases in seroprevalence following the emergence of novel variants, particularly Omicron, are consistent with the increased transmissibility and immune evasion of these variants [17].

Comparisons of seroprevalence between settings and groups are complicated by the extended period over which samples were taken, and so we did not undertake formal comparisons. However, seroprevalence appeared to vary between settings, being lowest in Europe and highest in HIV-negative participants in SA and, within Europe, highest in the UK. This may reflect several factors, including age (the UK cohort was older than the other European cohorts, and studies have shown seroprevalence increases with age [18-20]), the timing of testing in relation to SARS-CoV-2 circulation in different settings, and variation in mitigation strategies between settings. Seroprevalence was higher among CALWHIV with CD4 counts ≥350 cells/μL than those with lower CD4 counts, consistent with other studies [21]. This could reflect an impaired serological response in those with lower CD4 count (lack of detectable response, lower peak antibody levels, or faster waning) or greater avoidance of social contact in those with lower CD4 counts. For example, early in the pandemic, the British HIV Association advised adult PLWHIV with a CD4 count <200 cells/μL, detectable VL or not on ART to strictly follow social distancing advice [22]. We did not see a difference in seroprevalence by viral suppression status (<50 copies/mL), although previous studies have reported higher seroprevalence among PLWHIV who were virologically suppressed [11, 23].

It is difficult to compare our results for CALWHIV with those of general population studies due to differences in key characteristics (e.g., age groups and sources of samples) and calendar period, as well as variation in SARS-CoV-2 dynamics in different settings and populations. Household serosurveys in SA reported seroprevalence ranging from 56% to ~80% in children and adolescents in October–December 2021 [19, 24]. This is broadly consistent with our results for the same time frame in SA: 72% among CALWHIV overall and 68% among unvaccinated CALWHIV, and 77% among all HIV-negative participants versus 74% in unvaccinated HIV-negative participants. In the UK, population-based studies reported a seroprevalence of 20% by April–June 2021 among 0–18 year-olds in England [25], somewhat lower than our estimates for the corresponding quarter of 34% overall and 28% in unvaccinated participants, respectively (likely reflecting the older age of our study participants).

Vaccine coverage was relatively low among study participants (≤50% in each of the three cohort groups by the end of the study period) but increased over time corresponding to vaccine availability. For example, in the UK, PLWHIV aged ≥16 years were eligible for vaccination from February 2021; younger PLWHIV became eligible with the rest of their age group (September 2021 for 12–15 year-olds, February 2022 for 5–11 year-olds) [26]. In SA, people aged 18–34 years were eligible for vaccination from September 2021 [27], and 12–17 year-olds from October 2021 [28], with PLWHIV being prioritized. Therefore the lower vaccine coverage in SA compared to Europe in our study probably reflects later access in SA.

In SA, vaccine coverage was similar among CALWHIV and HIV-negative participants. Published population data show 26.2% of 12–17 year-olds and 36.6% of 18–34 year-olds having received at least one dose by March 2022 [29], similar to our findings. Vaccine coverage (≥1 dose) among CALWHIV in Europe in our study (50%) was similar to coverage among 12–15 year-olds in the general population in England (53.5%) by August 2022 [30]. As in adults [31], coverage varied by factors such as ethnicity and deprivation [30], analysis of which is beyond the scope of this paper. It will be important to monitor uptake of vaccines among CALWHIV and better understand the causes including delayed access in high HIV burden settings and/or vaccine hesitancy, all of which are critical in informing future pandemic preparedness.

The higher seroprevalence among the HIV-negative participants compared to CALWHIV in SA, albeit with wide CIs, is consistent with previous results from SA [11], Mozambique [8], and the USA [32]. We also found that seroprevalence was lower in CALWHIV with CD4 counts ≥350 cells/μL than among HIV-negative participants. The authors of the US study proposed that PLWHIV may have been more careful to avoid infection [32], and presented evidence of a diminished IgG response to SARS-CoV-2 infection, both of which might apply in our study. Diminished antibody responses to childhood vaccines among CALWHIV, compared to HIV-exposed uninfected children, have also been reported [33].

This study has some important limitations. Our pragmatic approach to data collection meant that a variety of tests were used; the predominance of S-antibody tests means that, among the one-quarter of participants who were ever vaccinated, we cannot distinguish between infection- versus vaccine-induced antibodies. However, we also present estimates for unvaccinated participants and, where available, N-antibodies; 42% of those with N-antibody results were seropositive, indicating definite infection. Variations in antibody test sensitivity and specificity, and potentially antibody waning, mean that our seroprevalence estimates among the unvaccinated may be minimum estimates of the prevalence of previous SARS-CoV-2 infection (e.g., some participants who were seronegative at enrollment may have been previously seropositive). We combined data from Belgium, Greece, and Spain due to small numbers, potentially masking differences between countries. Although our data suggest that some CALWHIV revert from seropositive to seronegative, we cannot estimate the duration of antibody persistence; furthermore, cell-mediated immunity may provide protection and persist even in the absence of detectable antibody [34]. Finally, in this cohort of CALWHIV, largely comprising older adolescents with perinatally acquired HIV, almost all were on ART, most had undetectable VL and few were immunosuppressed, therefore our findings may not be generalizable to settings with younger cohorts and less well-controlled HIV.

We report a high seroprevalence of SARS-CoV-2 antibody among CALWHIV in Europe and SA, frequently in the absence of vaccination or reported prior infection/disease. We therefore infer that a high proportion of SARS-CoV-2 infections were mild or asymptomatic in this population. Further data on the extent and duration of protection conferred by infection- and vaccination-induced antibodies, and the relative importance of cell-mediated immunity, in this population are needed to better define their future susceptibility to (re-)infection and severe disease, and to optimize vaccination strategies.

## Supporting information

The European Pregnancy and Paediatric Infections Cohort Collaboration (EPPICC) SARS-CoV-2 Antibody Study Group supplementary material 1 The European Pregnancy and Paediatric Infections Cohort Collaboration (EPPICC) SARS-CoV-2 Antibody Study Group supplementary material

The European Pregnancy and Paediatric Infections Cohort Collaboration (EPPICC) SARS-CoV-2 Antibody Study Group supplementary material 2 The European Pregnancy and Paediatric Infections Cohort Collaboration (EPPICC) SARS-CoV-2 Antibody Study Group supplementary material

The European Pregnancy and Paediatric Infections Cohort Collaboration (EPPICC) SARS-CoV-2 Antibody Study Group supplementary material 3 The European Pregnancy and Paediatric Infections Cohort Collaboration (EPPICC) SARS-CoV-2 Antibody Study Group supplementary material

## Data Availability

The data that support the findings of this study are available on request from the corresponding author. The data are not publicly available due to their sensitive nature and privacy and ethical considerations.

## Notes

**Abbreviations**
ART: antiretroviral therapy
CALWHIV: children and adolescents living with HIV
CI: confidence interval
CTAAC: Cape Town Adolescent Antiretroviral Cohort
EPPICC: European Pregnancy and Paediatric Infections Cohort Collaboration
HIV: human immunodeficiency virus
IQR: interquartile range
ISARIC: International Severe Acute Respiratory and emerging Infection Consortium
MIS-C: multisystem inflammatory syndrome in children
PCR: polymerase chain reaction
SA: South Africa
UK: United Kingdom
VL: viral load
WHO: World Health Organization

## Supplementary material

The supplementary material for this article can be found at http://doi.org/10.1017/S0950268825000135.

## Project team

Charlotte Jackson (University College London, London, UK), Siobhan Crichton (University College London, London, UK), Alasdair Bamford (University College London, London, UK; Great Ormond Street Hospital for Children NHS Foundation Trust, London, UK), Arantxa Berzosa Sanchez (Hospital General Universitario “Gregorio Marañón”, Madrid, Spain), Kimberly C. Gilmour (Great Ormond Street Hospital for Children NHS Foundation Trust, London, UK; National Institute for Health Research Great Ormond Street Hospital Biomedical Research Centre, London, UK), Tessa Goetghebuer (Centre Hospitalier Universitaire St Pierre, Université libre de Bruxelles, Brussels, Belgium), Sarah May Johnson (Great Ormond Street Hospital for Children NHS Foundation Trust, London, UK), Ali Judd (University College London, London, UK; Fondazione Penta ETS, Padova, Italy), Antoni Noguera-Julian (Institut de Recerca Pediàtrica Sant Joan de Déu, Barcelona, Spain; Universitat de Barcelona, Barcelona, Spain; Red de Investigación Translacional en Infectología Pediátrica (RITIP), Madrid, Spain; CIBER de Epidemiología y Salud Pública (ISCIII), Madrid, Spain), Marthe Le Prevost (University College London, London, UK), Vana Spoulou (“Agia Sophia” Children’s Hospital, Athens, Greece), Kate Sturgeon (University College London, London, UK), Alla Volokha (Shupyk National Healthcare University of Ukraine, Kyiv, Ukraine), Heather J. Zar (University of Cape Town, Cape Town, South Africa), and Intira Jeannie Collins (senior author; University College London, London, UK).

## Writing committee, listed alphabetically

Nana Akua Asafu-Agyei (University of Cape Town, Cape Town, South Africa), Emma Carkeek (University of Cape Town, Cape Town, South Africa), Elizabeth Chappell (University College London, London, UK), Danielle Chilton (Guy’s and St Thomas’ NHS Foundation Trust, London, UK), Katja Doerholt (St George’s University Hospitals NHS Foundation Trust), Caroline Foster (Imperial College Healthcare NHS Trust, London, UK), Marie Antoinette Frick (Hospital Vall d´Hebron, Barcelona, Spain), Elizabeth Hamlyn (King’s College Hospital NHS Foundation Trust, London, UK), Sally Hawkins (King’s College Hospital NHS Foundation Trust, London, UK), Julia Kenny (Guy’s and St Thomas’ NHS Foundation Trust, London, UK), Hermione Lyall (Imperial College Healthcare NHS Trust, London, UK), Paddy McMaster (Manchester University NHS Foundation Trust, Manchester, UK), Marisa Navarro (Hospital General Universitario “Gregorio Marañón”, Madrid, Spain), Katia Prime (St George’s University Hospitals NHS Foundation Trust), Steven B Welch (University Hospitals Birmingham NHS Foundation Trust, Birmingham, UK).

## EPPICC/penta co-ordinating team

Elizabeth Chappell, Siobhan Crichton, Intira Jeannie Collins, Giorgia Dalla Valle, Charlotte Duff, Carlo Giaquinto, Charlotte Jackson, Ali Judd, Laura Mangiarini, Edith Milanzi, Karen Scott.

## Collaborating cohorts

*Belgium*: Hopital St Pierre Cohort, Brussels: Tessa Goetghebuer, MD, PhD; Marc Hainaut, MD PhD; Wivine Tremerie, Research nurse; Marc Delforge, data manager.

*Spain*: CoRISPE-cat, Catalonia: CoRISPE-cat receives financial support from the Instituto de Salud Carlos III through the Red Temática de Investigación Cooperativa en Sida (grant numbers RED RIS RD06/0006/0035 yRD06/0006/0021). Members: Hospital Universitari Vall d’Hebron, Barcelona (Pere Soler-Palacín, Maria Antoinette Frick and Santiago Pérez-Hoyos (statistician)), Hospital Universitari del Mar, Barcelona (Núria López), Hospital Universitari Germans Trias i Pujol, Badalona (María Méndez, Clara Carreras), Hospital Universitari JosepTrueta, Girona (Borja Guarch), Hospital Universitari Arnau de Vilanova, Lleida (Teresa Vallmanya, Laura Minguell-Domingo), Hospital Universitari Joan XXIII, Tarragona (Olga Calavia), Consorci Sanitari del Maresme, Mataró (Lourdes García), Hospital General de Granollers (Maite Coll), Corporació Sanitària Parc Taulí, Sabadell (Valentí Pineda), Hospital Universitari Sant Joan, Reus (Neus Rius), Fundació Althaia, Manresa (Núria Rovira), Hospital Son Espases, Mallorca (Joaquín Dueñas) and Hospital Sant Joan de Déu, Esplugues (Clàudia Fortuny, Anna Gamell, Antoni Noguera-Julian).

*Spain*: CoRISPE-S and Madrid cohort:

*Receive funding from:* Estudio del análisis clínico-epidemiológico de la infección por el vih en niños y adolescentes, mujeres embarazadas y sus hijos a nivel nacional. Ministerio Sanidad. Proyect 202007PN0002

*Pediatrics Units*: María José Mellado, Luis Escosa, Milagros García Hortelano, Talía Sainz, Carlos Grasa, Paula Rodríguez (Hospital Universitario La Paz, Madrid); Pablo Rojo, Luis Prieto-Tato, Cristina Epalza, Alfredo Tagarro, Sara Domínguez, Álvaro Ballesteros (Hospital Universitario Doce de Octubre, Madrid); José Tomás Ramos, Marta Illán, Arantxa Berzosa, (Hospital Clínico San Carlos, Madrid); Sara Guillén, Beatriz Soto (Hospital Universitario de Getafe, Madrid); María Luisa Navarro, Jesús Saavedra, Mar Santos, David Aguilera, Begoña Santiago, Santiago Jimenez de Ory (Hospital Universitario Gregorio Marañón, Madrid); Amanda Bermejo (Hospital Universitario de Móstoles, Madrid); María Penín (Hospital Universitario Príncipe de Asturias de Alcalá de Henares, Madrid); Jorge Martínez (Hospital Infantil Universitario Niño Jesús, Madrid); Katie Badillo (Hospital Universitario de Torrejón, Madrid); Ana Belén Jiménez (Hospital Fundación Jiménez Díaz, Madrid); Adriana Navas (Hospital Universitario Infanta Leonor, Madrid); Eider Oñate (Hospital Universitario Donostia, Guipúzcoa); Itziar Pocheville (Hospital Universitario Cruces, Vizcaya); Elisa Garrote (Hospital Universitario Basurto, Vizcaya); Elena Colino, Olga Afonso (Hospital Insular Materno Infantil, Gran Canaria); Jorge Gómez Sirvent (Hospital Universitario Virgen de la Candelaria, Tenerife); Mónica Garzón, Vicente Román (Hospital General, Lanzarote); Raquel Angulo (Hospital de Poniente de El Ejido, Almería); Olaf Neth, Lola Falcón (Hospital Universitario Virgen del Rocío, Sevilla); Pedro Terol (Hospital Universitario Virgen de la Macarena, Sevilla); Juan Luis Santos, Álvaro Vázquez (Hospital Universitario Virgen de las Nieves, Granada); Begoña Carazo, Antonio Medina (Hospital Regional Universitario, Málaga); Francisco Lendínez, Mercedes Ibáñez (Complejo Hospitalario Torrecárdenas, Almería); Estrella Peromingo, María Isabel Sánchez (Hospital Universitario Puerta del Mar, Cádiz); Beatriz Ruiz (Hospital Universitario Reina Sofía de Córdoba); Ana Grande (Complejo Hospitalario Universitario Infanta Cristina, Badajoz); Francisco José Romero (Complejo Hospitalario, Cáceres); Carlos Pérez, Alejandra Méndez (Hospital de Cabueñes, Asturias); Laura Calle (Hospital Universitario Central de Asturias); Marta Pareja (Complejo Hospitalario Universitario, Albacete); Begoña Losada (Hospital Virgen de la Salud, Toledo); Mercedes Herranz (Hospital Virgen del Camino, Navarra); Matilde Bustillo (Hospital Universitario Miguel Servet, Zaragoza); Pilar Collado (Hospital Clínico Universitario Lozano Blesa, Zaragoza); José Antonio Couceiro (Complejo Hospitalario Universitario, Pontevedra); Leticia Vila (Complejo Hospitalario Universitario, La Coruña); Consuelo Calviño (Hospital Universitario Lucus Augusti, Lugo); Ana Isabel Piqueras, Manuel Oltra (Hospital Universitario La Fe, Valencia); César Gavilán (Hospital Universitario de San Juan de Alicante, Alicante); Elena Montesinos (Hospital General Universitario, Valencia); Marta Dapena (Hospital General, Castellón); Beatriz Jiménez (Hospital Universitario Marqués de Valdecilla, Cantabria); Ana Gloria Andrés (Complejo Hospitalario, León); Víctor Marugán, Carlos Ochoa (Complejo Hospitalario, Zamora); Ana Isabel Menasalvas, Eloísa Cervantes, Beatriz Álvarez (Hospital Universitario Virgen de la Arrixaca, Murcia) and Paediatric HIV-BioBank integrated in the Spanish AIDS Research Network and collaborating Centers.

*Adults Units:* Cristina Díez (Hospital Universitario Gregorio Marañón, Madrid). Ignacio Bernardino, María Luisa Montes, Eulalia Valencia, Ana Delgado (Hospital Universitario La Paz, Madrid); Rafael Rubio, Federico Pulido, Otilia Bisbal (Hospital Universitario Doce de Octubre, Madrid); Alfonso Monereo Alonso (Hospital Universitario de Getafe, Madrid); Juan Berenguer, Cristina Díez, Teresa Aldamiz, Francisco Tejerina, Juan Carlos Bernaldo de Quirós, Belén Padilla, Raquel Carrillo, Pedro Montilla, Elena Bermúdez, Maricela Valerio (Hospital Universitario Gregorio Marañón, Madrid); Jose Sanz (Hospital Universitario Príncipe de Asturias de Alcalá de Henares, Madrid); Alejandra Gimeno (Hospital Universitario de Torrejon, Madrid); Miguel Cervero, Rafael Torres (Hospital Universitario Severo Ochoa de Leganés, Madrid); Santiago Moreno, María Jesús Perez, Santos del Campo (Hospital Universitario Ramon y Cajal, Madrid); Pablo Ryan, Jesus Troya (Hospital Universitario Infanta Leonor, Madrid); Jesus Sanz (Hospital Universitario La Princesa, Madrid); Juan Losa, Rafael Gomez (Hospital Universitario Fundacion Alcorcon, Madrid); Miguel Górgolas (Hospital Fundacion Jimenez Diaz, Madrid); Alberto Díaz, Sara de la Fuente (Hospital Universitario Puerta de Hierro de Majadahonda, Madrid); Jose Antonio Iribarren, Marıa Jose Aramburu, Lourdes Martinez (Hospital Universitario Donostia, Guipuzcoa); Ane Josune Goikoetxea (Hospital Universitario Cruces, Vizcaya); Sofia Ibarra, Mireia de la Peña (Hospital Universitario Basurto, Vizcaya); Víctor Asensi (Hospital Universitario Central de Asturias); Michele Hernandez (Hospital Universitario Insular, Gran Canaria); María Remedios Alemán, Ricardo Pelazas, María del Mar Alonso, Ana María López, Dácil García, Jehovana Rodriguez (Hospital Universitario de Canarias, Tenerife); Miguel Angel Cardenes (Hospital Universitario Doctor Negrin, Gran Canaria); Manuel A. Castaño, Francisco Orihuela, Inés Pérez, Mª Isabel Mayorga (Hospital Regional Universitario, Málaga); Luis Fernando Lopez-Cortes, Cristina Roca, Silvia Llaves (Hospital Universitario Virgen del Rocio, Sevilla); Marıa Jose Rios, Jesus Rodriguez, Virginia Palomo (Hospital Universitario Virgen de la Macarena, Sevilla); Juan Pasquau, Coral Garcia (Hospital Universitario Virgen de las Nieves, Granada); Jose Hernandez, Clara Martinez (Hospital Universitario Clinico San Cecilio, Granada); Antonio Rivero, Angela Camacho (Hospital Universitario Reina Sofia, Cordoba); Dolores Merino, Miguel Raffo, Laura Corpa (Hospital Universitario Juan Ramon Jimenez, Huelva); Elisa Martinez, Fernando Mateos, Jose Javier Blanch (Complejo Hospitalario Universitario, Albacete); Miguel Torralba (Hospital Universitario, Guadalajara); Piedad Arazo, Gloria Samperiz (Hospital Universitario Miguel Servet, Zaragoza); Celia Miralles, Antonio Ocampo, Guille Pousada (Hospital Alvaro Cunqueiro, Pontevedra); Alvaro Mena (Complejo Hospitalario Universitario, La Coruna); Marta Montero, Miguel Salavert, (Hospital Universitario La Fe, Valencia); Maria Jose Galindo, Natalia Pretel (Hospital Clinico Universitario, Valencia); Joaquín Portilla, Irene Portilla (Hospital General Universitario, Alicante); Felix Gutierrez, Mar Masia, Cati Robledano, Araceli Adsuar (Hospital General Universitario de Elche, Alicante); Carmen Hinojosa, Begoña Monteagudo (Hospital Clinico, Valladolid); Pablo Bachiller (Hospital General, Segovia); Jesica Abadía (Hospital Universitario Rio Hortega, Valladolid); Carlos Galera, Helena Albendin, Marian Fernandez (Hospital Universitario Virgen de la Arrixaca, Murcia); Jose Ramon Blanco (Complejo Hospitalario San Millan-San Pedro, la Rioja).

*Ukraine*: Paediatric HIV Cohort: Dr T. Kaleeva, Dr Y. Baryshnikova (Odessa Regional Centre for HIV/AIDS); Dr S. Soloha (Donetsk Regional Centre for HIV/AIDS); Dr N. Bashkatova (Mariupol AIDS Center); Dr I. Raus (Kyiv City Centre for HIV/AIDS); Dr O. Glutshenko, Dr Z. Ruban (Mykolaiv Regional Centre for HIV/AIDS); Dr N. Prymak (Kryvyi Rih); Dr G. Kiseleva (Simferopol); Dr Alla Volokha (Shupyk National Medical Academy of Postgraduate Education); Dr Ruslan Malyuta (Perinatal Prevention of AIDS Initiative, Odessa); Dr H. Bailey, Prof Claire Thorne (UCL, London, UK). Funding acknowledgment: PENTA Foundation.

*UK & Ireland*: Collaborative HIV Paediatric Study (CHIPS): CHIPS is funded by the NHS (London Specialised Commissioning Group) and has received additional support from Abbott, Boehringer Ingelheim, Bristol-Myers Squibb, GlaxoSmithKline, Gilead Sciences, Janssen and Roche. The MRC Clinical Trials Unit at UCL is supported by the Medical Research Council (https://www.mrc.ac.uk) program number MC_UU_00004/03.

*CHIPS Steering Committee:* Hermione Lyall (chair), Alasdair Bamford, Karina Butler, Katja Doerholt, Conor Doherty, Caroline Foster, Ian Harrison, Julia Kenny, Nigel Klein, Gillian Letting, Paddy McMaster, Fungai Murau, Edith Nsangi, Katia Prime, Andrew Riordan, Fiona Shackley, Delane Shingadia, Sharon Storey, Gareth Tudor-Williams, Anna Turkova, Steve Welch. *MRC Clinical Trials Unit:* Intira Jeannie Collins, Claire Cook, Siobhan Crichton, Donna Dobson, Keith Fairbrother, Diana M. Gibb, Ali Judd, Marthe Le Prevost, Nadine Van Looy. *Integrated Screening Outcome Surveillance Service (ISOSS)* **, UCL:** Helen Peters, Kate Francis, Claire Thorne.

*Hospitals participating in CHIPS in 2019/20:* University Hospitals Birmingham NHS Foundation Trust, Birmingham: L Thrasyvoulou, S Welch; Brighton and Sussex University Hospitals NHS Trust: K Fidler; University Hospitals Bristol NHS Foundation Trust, Bristol: J Bernatoniene, F Manyika; Calderdale and Huddersfield NHS Foundation Trust, Halifax: G Sharpe; Derby Teaching Hospitals NHS Foundation Trust: B Subramaniam; Glasgow Royal Hospital for Children, Glasgow: R Hague, V Price; Great Ormond Street Hospital for Children NHS Foundation Trust, London: J Flynn, N Klein, A Bamford, D Shingadia, K Grant, Karyn Moshal; Oxford University Hospitals NHS Foundation Trust, Oxford: S Yeadon, S Segal; King’s College Hospital NHS Foundation Trust, London: S Hawkins; Leeds Teaching Hospitals NHS Trust, Leeds: M Dowie; University Hospitals of Leicester NHS Trust, Leicester: S Bandi, E Percival; Luton and Dunstable Hospital NHS Foundation Trust, Luton: M Eisenhut; K Duncan; Milton Keynes General University Hospital NHS Foundation Trust, Milton Keynes: L Anguvaa, L Wren, Newcastle upon Tyne Hospitals NHS Foundation Trust, Newcastle: T Flood, A Pickering; The Pennine Acute Hospitals NHS Trust, Manchester: P McMaster, C Murphy; North Middlesex University Hospital NHS Trust, London: J Daniels, Y Lees; Northampton General Hospital NHS Trust, Northampton: F Thompson; London North West Healthcare NHS Trust, Middlesex; A Williams, B Williams, S Pope; Barts Health NHS Trust, London: Dr S Libeschutz; Nottingham University Hospitals NHS Trust, Nottingham: L Cliffe, S Southall; Portsmouth Hospitals NHS Trust, Portsmouth: A Freeman; Raigmore Hospital, Inverness: H Freeman; Royal Belfast Hospital for Sick Children, Belfast: S Christie; Royal Berkshire NHS Foundation Trust, Reading: A Gordon; Royal Children’s Hospital, Aberdeen: D Rosie Hague, L Clarke; Royal Edinburgh Hospital for Sick Children, Edinburgh: L Jones, L Brown; Royal Free NHS Foundation Trust, London: M Greenberg; Alder Hey Children’s NHS Foundation Trust, Liverpool: C Benson, A Riordan; Sheffield Children’s NHS Foundation Trust, Sheffield: L Ibberson, F Shackley; University Hospital Southampton NHS Foundation Trust, Southampton: S Patel, J Hancock; St George’s University Hospitals NHS Foundation Trust, London: K Doerholt, K Prime, M Sharland, S Storey; Imperial College Healthcare NHS Trust, London: EGH Lyall, C Foster, P Seery, G Tudor-Williams, N Kirkhope, S Raghunanan; Guy’s and St Thomas’ NHS Foundation Trust, London: Dr Julia Kenny, A Callaghan; University Hospitals of North Midlands NHS Trust, Stoke On Trent: A Bridgwood, P McMaster; University Hospital of Wales, Cardiff: J Evans, E Blake; NHS Frimley Health Foundation Trust, Slough: A Yannoulias.

*South Africa (CTAAC)*: We thank Prof Landon Myer for his role in establishing CTAAC and Dr Lisa Frigati for advice on this study; we acknowledge the CTACC clinical, data and laboratory teams for their work on this study.
