# Supplementary material for: Are children and adolescents living with HIV in Europe and South Africa at higher risk of SARS-CoV-2 and poor COVID-19 outcomes?
Source: Epidemiol Infect. 2025 Feb 21;153:e44. doi: 10.1017/S0950268825000135 (PMC11951227; doi:10.1017/S0950268825000135)
Supplement: The European Pregnancy and Paediatric Infections Cohort Collaboration (EPPICC) SARS-CoV-2 Antibody Study Group supplementary material 1 — The European Pregnancy and Paediatric Infections Cohort Collaboration (EPPICC) SARS-CoV-2 Antibody Study Group supplementary material [file S0950268825000135sup001.pdf]

EPPICC-COVID: COVID-19 CASE REPORT FORM (final version 3.0)

Adapted from WHO/ISARIC forms

COHORT: PATIENT ID:

**Section 1. Inclusion criteria, meeting <u>one or more</u> of the following:**
COVID-19 positive PCR test result (give details in Section 2) ☐<br><br>AND/OR<br><br>Hospitalised, with no other (non-COVID-19) eventual diagnosis, and with one or more of:<br>• A history of self-reported feverishness or measured fever >38°C ☐<br>• Cough ☐<br>• Dyspnoea (shortness of breath) or tachypnoea (increased respiratory rate) ☐<br>• Clinical suspicion of COVID-19 despite not meeting other criteria ☐

Section 2. COVID-19 PCR testing (please report all available results) and admission to hospital

PCR test 1	Reason for test: Contact tracing ☐<br>Routine screening ☐ Research study ☐<br>Symptomatic ☐ Other ☐ Unknown ☐<br>If other, specify:<br><br>Date:<br><br>Result (categorical): Positive ☐<br>Negative ☐ Indeterminate ☐<br><br>Result (quantitative, including units):	PCR test 2	Reason for test: Contact tracing ☐<br>Routine screening ☐ Research study ☐<br>Symptomatic ☐ Other ☐ Unknown ☐<br>If other, specify:<br><br>Date:<br><br>Result (categorical): Positive ☐<br>Negative ☐ Indeterminate ☐<br><br>Result (quantitative, including units):
PCR test 3	Reason for test: Contact tracing ☐<br>Routine screening ☐ Research study ☐<br>Symptomatic ☐ Other ☐ Unknown ☐<br>If other, specify:<br><br>Date:<br><br>Result (categorical): Positive ☐<br>Negative ☐ Indeterminate ☐<br><br>Result (quantitative, including units):	PCR test 4	Reason for test: Contact tracing ☐<br>Routine screening ☐ Research study ☐<br>Symptomatic ☐ Other ☐ Unknown ☐<br>If other, specify:<br><br>Date:<br><br>Result (categorical): Positive ☐<br>Negative ☐ Indeterminate ☐<br><br>Result (quantitative, including units):

Admitted to hospital	Yes ☐ No ☐ Unknown ☐<br>If yes, date of admission:

Section 3. Vital signs at admission *(for those hospitalised only, and if not hospitalised then skip to section 4)*

Temperature (°C)		Systolic blood pressure (mmHg)	
Heart rate (beats/minute)		Diastolic blood pressure (mmHg)	

Respiratory rate (breaths/minute)			
Severe dehydration	Yes ☐ No ☐ Unknown ☐	Sternal capillary refill time >2 secs	Yes ☐ No ☐ Unknown☐
Oxygen saturation (%)	on room air ☐ oxygen therapy ☐ unknown ☐		
Conscious state	Alert ☐ Response to verbal stimuli ☐ Response to painful stimuli ☐ Unresponsive ☐ Unknown ☐		
Mid-upper arm circumference (mm)			
Height (cm)		Weight (kg)	

Section 4. Signs and symptoms ***(at admission for those hospitalised, at time diagnosed for those not hospitalised).***

Any symptoms experienced	Yes ☐ No ☐ Unknown ☐<br>If yes, date of symptom onset:<br><br>*If no, skip to section 9. If unknown, skip to section 5.*		
History of fever	☐	Increased respiratory rate	☐
Cough	☐	Lower chest indrawing	☐
with sputum production	☐	Headache	☐
with haemoptysis	☐	Altered consciousness/ confusion	☐
Sore throat	☐	Seizures	☐
Runny nose	☐	Abdominal pain	☐
Wheezing	☐	Vomiting/nausea	☐
Chest pain	☐	Diarrhoea	☐
Muscle aches	☐	Conjunctivitis	☐
Joint pain (arthralgia)	☐	Skin rash	☐
Fatigue/malaise	☐	Skin ulcers	☐
Loss of taste	☐	Lymphadenopathy	☐
Loss of smell	☐	Inability to walk	☐
Shortness of breath	☐	Other	☐<br>If yes, specify:

Section 5. Diagnostic/pathogen testing ***(at any point during admission for those hospitalised, and at any point during COVID-19 illness for those not hospitalised)***

Chest x-ray/chest CT performed	Yes ☐ No ☐ Unknown ☐<br>If yes: Normal ☐ Abnormal ☐ Unknown ☐<br>If abnormal, findings:
Any respiratory pathogen (excluding SARS-CoV-2)	Positive ☐ Negative ☐ Not done ☐<br>If positive, specify:

COHORT: PATIENT ID:

Falciparum malaria	Positive ☐ Negative ☐<br>Not done☐	Non-falciparum malaria	Positive ☐ Negative ☐<br>Not done ☐

Section 6. Complications ***(at any point during admission for those hospitalised, if not hospitalised then skip to section 7)***

Were any complications experienced?	Yes ☐ No ☐ Unknown ☐<br>*If yes, tick all that apply below. If no/unknown, skip to section 7.*		
Shock	☐	Bleeding	☐
Seizure	☐	Endocarditis	☐
Meningitis/encephalitis	☐	Myocarditis/pericarditis	☐
Anaemia	☐	Acute renal injury	☐
Cardiac arrhythmia	☐	Pancreatitis	☐
Cardiac arrest	☐	Liver dysfunction	☐
Pneumonia	☐	Cardiomyopathy	☐
Bronchitis	☐	Venous thrombosis	☐
Acute respiratory distress syndrome (ARDS)	☐	Arterial thrombosis	☐
Stroke: ischaemic stroke	☐	Colitis	☐
Stroke: intracerebral haemorrhage	☐	Uveitis	☐
Bacteraemia	☐	Appendicitis	☐
Other	☐<br>Specify:		

Section 7. Medications ***(at any point during COVID-19 illness)***

Were any medications received?	Yes ☐ No ☐ Unknown ☐<br>*If yes, give details below. If no/unknown, skip to section 8*		
Oral/orogastric fluids	Yes ☐ No ☐	Intravenous fluids	Yes ☐ No ☐
Treatments for COVID-19 (including antivirals, immunomodulators but excluding coriticosteroids).<br><br>Do not include antiretrovirals used as part of ongoing HIV medication.	Yes ☐ No ☐<br>If yes: Ribavirin ☐ Lopinavir/ritonavir ☐ Neuraminidase inhibitor ☐ Tocilizumab ☐ Anakinra ☐ Ivermectin ☐ Interferon alpha ☐ Interferon beta ☐ Remdesivir ☐ Other ☐<br><br>If other, specify agent:		
Corticosteroid	Yes ☐ No ☐<br>If yes, specify agent:	Other experimental agent	Yes ☐ No ☐<br>If yes, specify agent:
Antibiotic	Yes ☐ No ☐<br>If yes, specify agent:	Non-steroidal anti-inflammatory (NSAID)	Yes ☐ No ☐<br>If yes, specify agent:

COHORT: PATIENT ID:

Antifungal agent	Yes ☐ No ☐<br>If yes, specify agent:	Systemic anticoagulation	Yes ☐ No ☐<br>If yes, specify agent:
Antimalarial agent	Yes ☐ No ☐<br>If yes, specify agent:		
Were any medications received as part of a COVID-19 clinical trial?	Yes ☐ No ☐ Unknown ☐<br>If yes, specify agent:		
**Section 8. Supportive care** ***(at any point during admission for those hospitalised, if not hospitalised then skip to Section 9)***			
Was any additional supportive care received?	Yes ☐ No ☐ Unknown ☐<br>*If yes, give details below. If no or unknown, skip to section 9.*		
ICU or high dependency unit admission	Yes ☐ No ☐<br>If yes,<br>total duration (days):<br>date of ICU admission:<br>date of ICU discharge/death:	Oxygen therapy	Yes ☐ No ☐<br>If yes,<br>total duration (days):<br>interface (tick all that apply):<br>nasal prongs ☐<br>HF nasal cannula ☐ mask ☐<br>mask with reservoir ☐<br>CPAP/NIV mask ☐
Non-invasive ventilation (e.g. BIPAP, CPAP)	Yes ☐ No ☐<br>If yes, total duration (days):	Invasive ventilation (any)	Yes ☐ No ☐<br>If yes, total duration (days):
Extracorporeal (ECMO) support	Yes ☐ No ☐<br>If yes, total duration (days):	Prone position	Yes ☐ No ☐<br>If yes, total duration (days):
Inotropes/ vasopressors	Yes ☐ No ☐<br>If yes, total duration (days):	Renal replacement therapy (RRT) or dialysis	Yes ☐ No ☐<br>If yes, total duration (days):
**Section 9. COVID-19 disease severity (worst disease severity at any point during disease course)**			
What was the worst disease severity experienced based on the WHO disease severity definition (see criteria at the bottom of form):<br><br>Asymptomatic ☐ Mild ☐ Moderate ☐ Severe ☐ Critical ☐			

COHORT: PATIENT ID:

Section 10. Outcome

Among hospitalised patients:
Outcome: Discharged ☐ Still hospitalised ☐ Palliative discharge ☐ Death ☐ Unknown ☐
If discharged, date of discharge:
If died, date of death:

Long term outcomes for hospitalised and non-hospitalised patients:
Were symptoms ongoing at 1 month after first onset of symptoms?
Yes ☐ No ☐ Unknown ☐
Were symptoms ongoing at 3 months after first onset of symptoms?
Yes ☐ No ☐ Unknown ☐
Were symptoms ongoing at 6 months after first onset of symptoms?
Yes ☐ No ☐ Unknown ☐

If yes symptoms ongoing, please describe:

Date form completed:

WHO definition of COVID-19 disease severity:

WHO Clinical classification	Based on available clinical records	Based on self-report, if clinical records are not available
Mild	No hypoxia or pneumonia	Did not receive oxygen
Moderate	Clinical signs of non-severe pneumonia *AND* SpO2>90% on room air	-
Severe	Adults/adolescents:<br>• Clinical signs of severe pneumonia *AND* SpO2 <90% on room air; OR<br>• RR > 30 breaths/min<br><br>Children: Clinical signs of severe pneumonia *AND* at least one of the following:<br>• central cyanosis; OR<br>• SpO2 < 90%; OR<br>• severe respiratory distress (e.g. fast breathing, grunting, very severe chest indrawing); OR<br>• general danger sign(s) (inability to breastfeed or drink, lethargy or unconsciousness, convulsions)	Received oxygen (or told you they needed it, but it was not available)
Critical	• ARDS; OR<br>• sepsis/septic shock; OR<br>• pulmonary embolism, acute coronary syndrome, acute stroke; OR<br>• Multi-Inflammatory Syndrome in Children and adolescents temporally related to COVID-19	Received invasive ventilation (or max available respiratory support)
