# Supplementary material for: Are children and adolescents living with HIV in Europe and South Africa at higher risk of SARS-CoV-2 and poor COVID-19 outcomes?
Source: Epidemiol Infect. 2025 Feb 21;153:e44. doi: 10.1017/S0950268825000135 (PMC11951227; doi:10.1017/S0950268825000135)
Supplement: The European Pregnancy and Paediatric Infections Cohort Collaboration (EPPICC) SARS-CoV-2 Antibody Study Group supplementary material 2 — The European Pregnancy and Paediatric Infections Cohort Collaboration (EPPICC) SARS-CoV-2 Antibody Study Group supplementary material [file S0950268825000135sup002.pdf]

# EPPICC-COVID: MIS-C CASE REPORT FORM (final version 3.0)

Adapted from WHO/ISARIC forms

COHORT: PATIENT ID:

| **Section 1. Inclusion criteria, meeting <u>each</u> of the following definition of multi-system inflammation syndrome in children (MIS-C):** |
|---|
| Aged 0-19 years and fever ≥3 days ☐<br><br>AND<br><br>Elevated markers of inflammation (e.g. ESR, CRP) ☐<br><br>AND<br><br>No other obvious microbial cause of inflammation ☐<br><br>AND<br><br>Evidence of COVID-19 (PCR or antigen or serology) or likely contact with COVID case ☐<br><br>AND<br><br>At least two of:<br>a) rash or conjunctivitis or muco-cutaneous inflammation signs ☐<br>b) hypotension or shock ☐<br>c) features of myocardial dysfunction or pericarditis, or valvulitis, or coronary abnormalities ☐<br>d) evidence of coagulopathy ☐<br>e) acute gastrointestinal problems ☐ |

| **Section 2. COVID-19 PCR and antibody testing (please report all available results), date of symptom onset and hospital admission** | | | |
|---|---|---|---|
| Date of onset of first symptom or sign of COVID-19 | | | |
| Date of first suspicion of MIS-C | | | |
| Date of admission to hospital | | | |
| PCR test 1 | Reason for test: Contact tracing ☐ Routine screening ☐ Research study ☐ Symptomatic ☐ Other ☐ Unknown ☐<br>If other, specify:<br><br>Date:<br><br>Result (categorical): Positive ☐ Negative ☐ Indeterminate ☐<br><br>Result (quantitative, including units): | PCR test 2 | Reason for test: Contact tracing ☐ Routine screening ☐ Research study ☐ Symptomatic ☐ Other ☐ Unknown ☐<br>If other, specify:<br><br>Date:<br><br>Result (categorical): Positive ☐ Negative ☐ Indeterminate ☐<br><br>Result (quantitative, including units): |

COHORT: PATIENT ID:

| | | | |
|---|---|---|---|
| PCR test 3 | Reason for test: Contact tracing ☐ Routine screening ☐ Research study ☐ Symptomatic ☐ Other ☐ Unknown ☐<br>If other, specify:<br><br>Date:<br><br>Result (categorical): Positive ☐ Negative ☐ Indeterminate ☐<br><br>Result (quantitative, including units): | PCR test 4 | Reason for test: Contact tracing ☐ Routine screening ☐ Research study ☐ Symptomatic ☐ Other ☐ Unknown ☐<br>If other, specify:<br><br>Date:<br><br>Result (categorical): Positive ☐ Negative ☐ Indeterminate ☐<br><br>Result (quantitative, including units): |
| Antibody test 1 | Reason for test: Contact tracing ☐ Routine screening ☐ Research study ☐ Symptomatic ☐ Other ☐ Unknown ☐<br>If other, specify:<br><br>Type: Serology IgM ☐ Serology IgG ☐ Total IgM/IgG ☐ Other ☐<br>If other type, please specify:<br><br>Brand: Abbot ☐ Siemens ☐ EDI ☐ Ortho ☐ Other ☐<br>If other brand, please specify:<br><br>Date:<br><br>Result (categorical): Positive ☐ Negative ☐ Indeterminate ☐<br><br>Result (quantitative, including units): | Antibody test 2 | Reason for test: Contact tracing ☐ Routine screening ☐ Research study ☐ Symptomatic ☐ Other ☐ Unknown ☐<br>If other, specify:<br><br>Type: Serology IgM ☐ Serology IgG ☐ Total IgM/IgG ☐ Other ☐<br>If other type, please specify:<br><br>Brand: Abbot ☐ Siemens ☐ EDI ☐ Ortho ☐ Other ☐<br>If other brand, please specify:<br><br>Date:<br><br>Result (categorical): Positive ☐ Negative ☐ Indeterminate ☐<br><br>Result (quantitative, including units): |

| | | | |
|---|---|---|---|
| Antibody test 3 | Reason for test: Contact tracing ☐ Routine screening ☐ Research study ☐ Symptomatic ☐ Other ☐ Unknown ☐<br>If other, specify:<br><br>Type: Serology IgM ☐ Serology IgG ☐ Total IgM/IgG ☐ Other ☐<br>If other type, please specify:<br><br>Brand: Abbot ☐ Siemens ☐ EDI ☐ Ortho ☐ Other ☐<br>If other brand, please specify:<br><br>Date:<br><br>Result (categorical): Positive ☐ Negative ☐ Indeterminate ☐<br><br>Result (quantitative, including units): | Antibody test 4 | Reason for test: Contact tracing ☐ Routine screening ☐ Research study ☐ Symptomatic ☐ Other ☐ Unknown ☐<br>If other, specify:<br><br>Type: Serology IgM ☐ Serology IgG ☐ Total IgM/IgG ☐ Other ☐<br>If other type, please specify:<br><br>Brand: Abbot ☐ Siemens ☐ EDI ☐ Ortho ☐ Other ☐<br>If other brand, please specify:<br><br>Date:<br><br>Result (categorical): Positive ☐ Negative ☐ Indeterminate ☐<br><br>Result (quantitative, including units): |

**Section 3. Vital signs at diagnosis of MIS-C**

| | | | |
|---|---|---|---|
| Temperature (°C) | | Systolic blood pressure (mmHg) | |
| Heart rate (beats/minute) | | Diastolic blood pressure (mmHg) | |
| Respiratory rate (breaths/minute) | | | |
| Severe dehydration | Yes ☐ No ☐ Unknown ☐ | Sternal capillary refill time >2 secs | Yes ☐ No ☐ Unknown ☐ |
| Oxygen saturation (%) | on room air ☐ oxygen therapy ☐ unknown ☐ | | |
| Conscious state | Alert ☐ Response to verbal stimuli ☐ Response to painful stimuli ☐ Unresponsive ☐ Unknown ☐ | | |
| Mid-upper arm circumference (mm) | | | |
| Height (cm) | | Weight (kg) | |

**Section 4. Summary of clinical features, at any point during admission**

| | | | |
|---|---|---|---|
| Bilateral conjunctivitis | Yes, purulent ☐<br>Yes, non-purulent ☐<br>No ☐ | Rash | Yes ☐ No ☐<br>If yes, type of rash: |
| Fever | Yes ☐ No ☐ | Oral mucosal inflammation signs | Yes ☐ No ☐ |
| Peripheral cutaneous inflammation signs (hands or feet) | Yes ☐ No ☐ | Urinary output <2 mL/kg/hr | Yes ☐ No ☐ |

COHORT: PATIENT ID:

| | | | |
|---|---|---|---|
| Hypotension (age-appropriate) | Yes ☐ No ☐ | Chest pain | Yes ☐ No ☐ |
| Tachycardia (age-appropriate) | Yes ☐ No ☐ | Tachypnoea (age-appropriate) | Yes ☐ No ☐ |
| Prolonged capillary refill time | Yes ☐ No ☐ | Respiratory distress | Yes ☐ No ☐ |
| Pale/mottled skin | Yes ☐ No ☐ | Abdominal pain | Yes ☐ No ☐ |
| Cold hands/feet | Yes ☐ No ☐ | Diarrhoea | Yes ☐ No ☐ |
| Vomiting | Yes ☐ No ☐ | Headache | Yes ☐ No ☐ |
| Confusion | Yes ☐ No ☐ | Seizures | Yes ☐ No ☐ |
| Encephalopathy | Yes ☐ No ☐ | Neck pain | Yes ☐ No ☐ |
| Other | Yes ☐ No ☐<br>If yes, specify: | | |

**Section 5. Imaging/pathogen testing, at any point during admission**

| | | | |
|---|---|---|---|
| Chest x-ray | Yes ☐ No ☐ Unknown ☐<br>If yes, findings: | Chest CT | Yes ☐ No ☐ Unknown ☐<br><br>If yes,<br>Were infiltrates present:<br>Yes ☐ No ☐ Unknown ☐<br><br>Other findings: |
| Echocardiography | Yes ☐ No ☐ Unknown ☐<br><br>If yes,<br>Date of most recent abnormal echocardiogram:<br><br>Were there features of,<br>myocardial dysfunction:<br>Yes ☐ No ☐ Unknown ☐<br>pericarditis:<br>Yes ☐ No ☐ Unknown ☐<br>valvulitis:<br>Yes ☐ No ☐ Unknown ☐<br>coronary artery dilation:<br>Yes ☐ No ☐ Unknown ☐ | Electrocardiogram (ECG) | Yes ☐ No ☐ Unknown ☐<br><br>If yes,<br>Date of most recent abnormal ECG:<br><br>What were the findings: |

| | | | |
|---|---|---|---|
| Other cardiac imaging | Yes ☐ No ☐ Unknown ☐<br><br>If yes,<br>Name of imaging:<br><br>Most abnormal results: | Bacterial pathogen testing | Positive ☐ Negative ☐ Not done ☐<br>If positive, specify: |
| Abdominal imaging | Yes ☐ No ☐ Unknown ☐<br>If yes:<br><br>Name of imaging:<br><br>Findings: | Brain imaging | Yes ☐ No ☐ Unknown ☐<br>If yes:<br><br>Name of imaging:<br><br>Findings: |
| Electroencephalogram (EEG) | Yes ☐ No ☐ Unknown ☐<br>If yes, findings: | | |
| **Section 6. Treatment, at any point during admission** | | | |
| Oral/orogastric fluids | Yes ☐ No ☐ Unknown ☐ | Intravenous fluids | Yes ☐ No ☐ Unknown ☐ |
| Treatments for COVID-19 (including antivirals, immunomodulators but excluding coriticosteroids).<br><br>Do not include antiretrovirals used as part of ongoing HIV medication. | Yes ☐ No ☐ Unknown ☐<br><br>If yes: Ribavirin ☐ Lopinavir/ritonavir ☐ Neuraminidase inhibitor ☐ Tocilizumab ☐ Anakinra ☐ Ivermectin ☐ Interferon alpha ☐ Interferon beta ☐ Remdesivir ☐ Other ☐<br><br>If other, specify: | | |
| Corticosteroid (not topical) | Yes ☐ No ☐ Unknown ☐<br>If yes, specify agent: | Antimalarial | Yes ☐ No ☐ Unknown ☐<br>If yes, specify agent: |
| IV immune globulin | Yes ☐ No ☐ Unknown ☐<br>If yes, specify agent: | Other experimental agent | Yes ☐ No ☐ Unknown ☐<br>If yes, specify agent: |
| Non-steroidal anti-inflammatory (NSAID) | Yes ☐ No ☐ Unknown ☐<br>If yes, specify agent: | Antibiotic | Yes ☐ No ☐ Unknown ☐<br>If yes, specify agent: |

| | | | |
|---|---|---|---|
| Antifungal | Yes ☐ No ☐ Unknown ☐<br>If yes, specify agent: | Systemic anticoagulation | Yes ☐ No ☐ Unknown ☐<br>If yes:<br>Prophylaxis ☐ Treatment ☐<br>Specify agent: |
| Other | Yes ☐ No ☐ Unknown ☐<br>If yes, specify agent: | | |
| Were any medications received as part of a COVID-19 clinical trial? | Yes ☐ No ☐ Unknown ☐<br>If yes, specify: | | |
| **Section 7. Supportive care, at any point during admission** | | | |
| ICU or high dependency unit admission | Yes ☐ No ☐ Unknown ☐<br>If yes,<br>total duration (days):<br>date of ICU admission:<br>date of ICU discharge/death: | Oxygen therapy | Yes ☐ No ☐ Unknown ☐<br>If yes,<br>total duration (days):<br>interface (tick all that apply):<br>nasal prongs ☐<br>HF nasal cannula☐<br>mask ☐ mask with reservoir☐<br>CPAP/NIV mask ☐ |
| Non-invasive ventilation (e.g. BIPAP, CPAP) | Yes ☐ No ☐ Unknown ☐<br>If yes, total duration (days): | Invasive ventilation (any) | Yes ☐ No ☐ Unknown ☐<br>If yes, total duration (days): |
| Extracorporeal (ECMO) support | Yes ☐ No ☐ Unknown ☐<br>If yes, total duration (days): | Prone position | Yes ☐ No ☐ Unknown ☐<br>If yes, total duration (days): |
| Inotropes/vasopressors | Yes ☐ No ☐ Unknown ☐<br>If yes, total duration (days): | Renal replacement therapy (RRT) or dialysis | Yes ☐ No ☐ Unknown ☐<br>If yes, total duration (days): |
| Plasma exchange | Yes ☐ No ☐ Unknown ☐ | HFOV | Yes ☐ No ☐ Unknown ☐ |
| Blood transfusion | Yes ☐ No ☐ Unknown ☐ | | |
| **Section 8. Outcome, at discharge/death** | | | |

COHORT: PATIENT ID:

| |
|---|
| Date form completed:<br><br>Outcome: Discharged ☐ Still hospitalised ☐ Palliative discharge ☐ Death ☐ Unknown ☐<br><br>If discharged,<br>Date of discharge:<br><br>If died,<br>Date of death:<br><br>Long term outcomes:<br>Were symptoms ongoing at 1 month after first onset of symptoms?<br>Yes ☐ No ☐ Unknown ☐<br>Were symptoms ongoing at 3 months after first onset of symptoms?<br>Yes ☐ No ☐ Unknown ☐<br>Were symptoms ongoing at 6 months after first onset of symptoms?<br>Yes ☐ No ☐ Unknown ☐<br><br>If yes symptoms ongoing, please describe: |

| | |
|---|---|
| What was the physician's impression of the final diagnosis? | Multisystem inflammatory syndrome: Yes ☐ No ☐ Unknown ☐<br>Kawasaki disease: Yes ☐ No ☐ Unknown ☐<br>Atypical Kawasaki disease: Yes ☐ No ☐ Unknown ☐<br>Toxic shock syndrome: Yes ☐ No ☐ Unknown ☐<br>Other: |
