# Supplementary material for: Are children and adolescents living with HIV in Europe and South Africa at higher risk of SARS-CoV-2 and poor COVID-19 outcomes?
Source: Epidemiol Infect. 2025 Feb 21;153:e44. doi: 10.1017/S0950268825000135 (PMC11951227; doi:10.1017/S0950268825000135)
Supplement: The European Pregnancy and Paediatric Infections Cohort Collaboration (EPPICC) SARS-CoV-2 Antibody Study Group supplementary material 3 — The European Pregnancy and Paediatric Infections Cohort Collaboration (EPPICC) SARS-CoV-2 Antibody Study Group supplementary material [file S0950268825000135sup003.docx]

**Are children and adolescents living with HIV in Europe and South Africa at higher risk of SARS-CoV-2 and poor COVID-19 outcomes?**

**Supplementary material**

The European Pregnancy and Paediatric Infections Cohort Collaboration (EPPICC) SARS CoV-2 antibody study group

*Definition of COVID-19*

Consistent with the WHO definition in use at the beginning of the study (<https://www.who.int/publications/i/item/WHO-2019-nCoV-Clinical_CRF-2020.4>; no longer available), COVID-19 disease was defined as one or more of:

- History of self-reported feverishness or measured fever >38C
- Cough
- Dyspnoea or tachypnoea
- Clinical suspicion despite not meeting above criteria

*Definition of MIS-C*

Consistent with the WHO case definition for MIS-C (<https://www.who.int/publications/i/item/WHO-2019-nCoV-MIS_Children_CRF-2020.2>), participants were considered to have MIS-C if they met all of the following criteria:

- Aged 0-19 years and fever ≥3 days
- At least two of: a) rash or conjunctivitis or muco-cutaneous inflammation signs; b) hypotension or shock; c) features of myocardial dysfunction or pericarditis, or valvulitis, or coronary abnormalities; d) evidence of coagulopathy; e) acute gastrointestinal problems
- Elevated markers of inflammation (e.g. ESR, CRP)
- No other obvious microbial cause of inflammation
- Evidence of COVID (PCR or antigen or serology) or likely contact with COVID case

Supplementary Table S1: Types of antibody tests used.

|  |  | **Type of antibody, n (%)** | | | | |
| --- | --- | --- | --- | --- | --- | --- |
|  |  | IgG | IgM | Total IgG/IgM | Unknown | Total |
| **Viral protein** | **Spike** | 1234 (78) | 1 (50) | 19 (28) | 1 (8) | 1255 (76) |
|  | **Nucleocapsid** | 310 (20) | 1 (50) | 0 | 0 | 311 (19) |
|  | **Other** | 7 (<1) | 0 | 2 (3) | 0 | 9 (1) |
|  | **Unknown** | 28 (1) | 0 | 47 (69) | 11 (92) | 86 (5) |
|  | **Total** | 1579 (100) | 2 (100) | 68 (100) | 12 (100) | 1661 (100) |

Supplementary Table S2: Reported co-morbidities at enrolment amongst study participants.

| **Co-morbidity** | **Number (%)** | | |
| --- | --- | --- | --- |
|  | **Europe (N = 493)** | **SA, HIV+ (N = 307)** | **SA, HIV- (N = 103)** |
| Any co-morbidity (n = 493, 307, 103) | 58 (12) | 6 (2) | 4 (4) |
| ≥2 co-morbidities (n = 493, 307, 103) | 12 (2) | 0 | 0 |
| Type 1 diabetes (n = 489, 307, 103) | 0 | 0 | 0 |
| Type 2 diabetes (n = 489, 249, 130) | 0 | 0 | 0 |
| Asthma (n = 489, 249, 103) | 4 (1) | 1 (<1) | 0 |
| Chronic kidney disease (n = 489, 249, 103) | 1 (<1) | 0 | 0 |
| Chronic pulmonary disease (n = 489, 249, 103) | 6 (1) | 0 | 0 |
| Chronic cardiac disease (n = 489, 0, 0) | 0 | --- | --- |
| Chronic liver disease (n = 489, 249, 103) | 0 | 0 | 0 |
| Hypertension (n = 489, 249, 103) | 0 | 0 | 0 |
| Chronic neurological disease (n = 486, 249, 103) | 12 (2) | 0 | 0 |
| Asplenia (n = 489, 249, 103) | 0 | 0 | 0 |
| Sickle cell disease (n = 487, 249, 103) | 1 (<1) | 0 | 0 |
| Solid organ transplant (n = 489, 307, 103) | 0 | 0 | 0 |
| Cancer (n = 488, 249, 103) | 3 (1) | 0 | 0 |
| Chemotherapy (n = 489, 249, 103) | 1 (<1) | 0 | 0 |
| Stem cell transplant (n = 489, 307, 103) | 0 | 0 | 0 |
| Immunosuppressive therapy (n = 489, 0, 0) | 2 (<1) | --- | --- |
| Developmental delay (n = 484, 249, 103)* | 16 (3) | 0 | 0 |
| Primary immunodeficiency (n = 489, 0, 0) | 0 | --- | --- |
| Active or latent TB (n = 489, 69, 10) | 1 (<1) | 2 (3) | 0 |
| Malaria (n = 489, 0, 0) | 0 | --- | --- |
| HCV (n = 451, 249, 103) | 7 (2) | 0 | 0 |
| HBV (n = 396, 0, 0) | 2 (<1) | --- | --- |
| Other co-morbidity (n = 489, 22, 5) | 11 (2) | 3 (14) | 1 (20) |

* 8 classified as mild, 3 moderate, 3 severe, 2 severity unspecified.

Supplementary Table S3: Characteristics of participants who were seronegative for S-antibodies on their first test following vaccination (n = 11)

| Characteristic |  | Number (%) or Median [IQR] |
| --- | --- | --- |
| Time since vaccination (n = 10) | Median [IQR] | 103 [15-163] |
|  | <30 days | 4 (40) |
| CD4 count (cells/μL) | Median [IQR] | 617 [433-950] |
|  | <350, n (%) | 2 (18) |
| VL <50 copies/mL (n = 10) |  | 6 (60) |

Supplementary Table S4: Reversions from seropositive to seronegative amongst participants with two test results who were seropositive on their first test (n = 283)

|  | **CALWHIV** | | **HIV negative participants** | |
| --- | --- | --- | --- | --- |
|  | Total | Reversions,  n (%) | Total | Reversions,  n (%) |
| All participants | 222 | 44* (20) | 61 | 1 (2) |
| Excluding participants vaccinated between tests | 203 | 44* (22) | 51 | 1 (2) |

*At the time of the first test: median CD4 count 513 cells/μL [IQR 393-728], 31/42 (74%) were virologically suppressed below 50 copies/mL and 32/42 (76%) below 1000 copies/mL.

Supplementary Figure S1: Timing of first and second tests, and vaccinations (where vaccination date was reported), by cohort.

Supplementary Figure S2: Vaccine coverage by cohort group and calendar quarter of sampling. Estimates are shown for quarters in which ≥10 participants gave samples (number of samples shown above each data point) error bars show exact 95% confidence intervals.

Supplementary Figure S3: Percentage of serology tests that were positive in the UK, Ukraine and other EPPICC countries, by calendar quarter, overall (top) and amongst participants who were unvaccinated at the time of the test (bottom). Numbers show the denominator for each estimate.
